# Single-trial P300 classification algorithm based on centralized multi-person data fusion CNN

**DOI:** 10.3389/fnins.2023.1132290

**Published:** 2023-02-22

**Authors:** Pu Du, Penghai Li, Longlong Cheng, Xueqing Li, Jianxian Su

**Affiliations:** ^1^School of Integrated Circuit Science and Engineering, Tianjin University of Technology, Tianjin, China; ^2^China Electronics Cloud Brain Technology Co., Ltd., Tianjin, China

**Keywords:** convolutional neural networks, centralized collaborative BCI, multi-person data fusion, single-trial, P300 classification

## Abstract

**Introduction:**

Currently, it is still a challenge to detect single-trial P300 from electroencephalography (EEG) signals. In this paper, to address the typical problems faced by existing single-trial P300 classification, such as complex, time-consuming and low accuracy processes, a single-trial P300 classification algorithm based on multiplayer data fusion convolutional neural network (CNN) is proposed to construct a centralized collaborative brain-computer interfaces (cBCI) for fast and highly accurate classification of P300 EEG signals.

**Methods:**

In this paper, two multi-person data fusion methods (parallel data fusion and serial data fusion) are used in the data pre-processing stage to fuse multi-person EEG information stimulated by the same task instructions, and then the fused data is fed as input to the CNN for classification. In building the CNN network for single-trial P300 classification, the Conv layer was first used to extract the features of single-trial P300, and then the Maxpooling layer was used to connect the Flatten layer for secondary feature extraction and dimensionality reduction, thereby simplifying the computation. Finally batch normalisation is used to train small batches of data in order to better generalize the network and speed up single-trial P300 signal classification.

**Results:**

In this paper, the above new algorithms were tested on the Kaggle dataset and the Brain-Computer Interface (BCI) Competition III dataset, and by analyzing the P300 waveform features and EEG topography and the four standard evaluation metrics, namely Accuracy, Precision, Recall and F1-score,it was demonstrated that the single-trial P300 classification algorithm after two multi-person data fusion CNNs significantly outperformed other classification algorithms.

**Discussion:**

The results show that the single-trial P300 classification algorithm after two multi-person data fusion CNNs significantly outperformed the single-person model, and that the single-trial P300 classification algorithm with two multi-person data fusion CNNs involves smaller models, fewer training parameters, higher classification accuracy and improves the overall P300-cBCI classification rate and actual performance more effectively with a small amount of sample information compared to other algorithms.

## Introduction

Brain-Computer Interface is a new way of human-computer interaction, which provides a direct communication link between the brain and a computer or other external devices (McFarland and Wolpaw, 2011). The Event-Related potential (ERP) is a time-locked measure of electrical activity of the cerebral surface representing a distinct phase of cortical processing (Patel and Azzam, 2005), and it is an endogenous potential linked to a person’s reaction to some stimuli or specific events. Typical examples of ERP are N200 and P300. P300 (Sutton et al., 1967), which is a positive peak waveform displayed at about 300 ms after being evoked by a small probability event, is one of the most studied, widely used and most prominent components of ERP (David et al., 2020; Kirasirova et al., 2020).

P300 classification detection is the focus of P300-BCI research, and fast and accurate recognition is crucial to improving the performance of P300-BCI (Huang et al., 2022). The P300 usually exhibits a low signal-to-noise ratio (SNR) (Zhang et al., 2022). In order to highlight its time-locked component and minimize the background noise, P300-BCI demands collecting, aggregating and averaging data from multiple trials to obtain a reliable output (Liu et al., 2018), which is time consuming and inefficient. Therefore it is a great challenge to correctly classify P300 in a single-trial. Up to now, the accuracy records of the single-trial P300 classification algorithms are as follows: Krusienski’s average classification accuracy using stepwise linear discriminant analysis (SWLDA) is about 35%. Hoffmann’s average classification accuracy using Bayesian Linear Discriminant Analysis (BLDA) is about 60%. Blankertz applied Shrinking Linear Discriminant Analysis (SKLDA) and achieved an average classification accuracy of about 70%. Zhang adopted spatiotemporal discriminant analysis (STDA) and attained an average classification accuracy of about 61%. The average classification accuracy of the support vector machine (SVM) algorithm developed by Kaper reaches 64.56%. And that value of discriminative canonical pattern matching (DCPM) proposed by Xiao comes to 71.23%, demonstrating that DCPM significantly outperformed other traditional methods in single-trial P300 classification with smaller training sample (Xu et al., 2018, 2021; Xiao et al., 2019a,b, 2021; Wang et al., 2020). Ma et al. (2021) proposed a capsule network-based model that improved the detection accuracy of single-trial P300, however, the calculation became complicated due to the increase in size. Zhang et al. (2022) filtered the data with xDAWN to improve the signal-to-noise ratio of EEG signals, but the spatial filtering method required manual selection of significant features after feature extraction, and then classifying them. It is highly specific to particular factors; however, the algorithm is often complex and its accuracy is influenced by feature selection (Zhang et al., 2022).

Deep learning is end-to-end learning with a simple structure that can be ported to a variety of tasks with high classification accuracy but high requirements for sample data. Nowadays, deep learning methods have made great progress in EEG-based target detection technology (Li et al., 2021), and based on this, some scholars have proposed other approaches for P300 classification, such as transfer learning (Wei et al., 2020), EEG Data Fusion (Panwar et al., 2020), Incep A-EEGNet (Xu et al., 2022), Combined Classifier (Yu et al., 2021), Principal Component Analysis (PCA) (Li et al., 2020) etc. At present, Daniela used CNN (Cecotti and Graser, 2010) with a large number of training samples to obtain an average accuracy of 78.19% for single-trial P300 (De Venuto and Mezzina, 2021) classification; For multiple trial P300 classification, Gao et al. (2021), proposed learning invariant patterns based on a CNN and big EEG data with an average accuracy of 80%. Liu et al. (2021) proposed a machine learning model based on one-dimensional convolutional capsule network (1D CapsNet), which attained a classification accuracy around 80%.

Currently, single-person BCI systems often fail to achieve the desired results because of significant individual differences and erratic execution due to the physical condition of the subjects. P300 usually has different temporal and spatial feature information, and to solve the single-trial P300 detection problem, suitable signal processing and classification algorithms are required to extract discriminative information from single-trial data (Zheng et al., 2020). Existing P300-BCI classification algorithms do not extract sufficient spatial and temporal information at the data level in feature extraction, and data must be collected from multiple trials to obtain summary and average values. With the development of complex BCI systems, the concept of multi-person cBCI has been proposed to improve overall BCI performance by fusing brain activity obtained from multiple subjects. Wang and Jung (2011) demonstrated that cBCI can improve the performance of single-trial P300 measurements by fusing brain activity from multiple subjects. Zheng et al. (2020) introduced an cross-session EEG dataset to improve the performance and utility of a collaborative RSVP-based BCI system. Song et al. (2022) proposed a Mutual Learning Domain Adaptation Network (MLDANet) cBCI framework with information interaction, dynamic learning, and individual transfer capabilities that exhibited superior population detection performance. Li P. et al. (2022) applied migration learning-based CNNs to steady-state visual evoked potentials (SSVEP). Li C. et al. (2022) proposed a fourth-order cumulative volume feature extraction method (CUM4-CSP) based on the common spatial pattern (CSP) algorithm.

In terms of BCI systems, Tian and Wang (2019) developed a multi-brain collaboration-based BCI music therapy system to help people with disabilities enjoy music and receive rehabilitation training services in the arts. Zhang et al. (2021) compared different group sizes, variations in integration strategies and their effects on group performance. Liu (2022) proposed a concrete mapping model based on human perception of sound and aesthetic transformation from sound to visual expression, forming a design representation method for interactive sound visualization practice. Currently, multi-person cBCI systems are not widely used in interactive control (Miao et al., 2020). Therefore, the research in this field can promote the development of BCI technology (Gu et al., 2021; Zhang et al., 2021).

**FIGURE 1:**
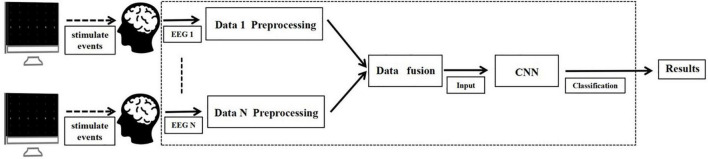
Structure of centralized collaborative brain-computer interfaces (cBCI) system.

Current research divides cBCI into two paradigms, namely distributed cBCI and centralized cBCI systems (Wang and Jung, 2011; Li P. et al., 2022). In distributed cBCI, the EEG information of the subjects is collected separately through the corresponding BCI subsystems for subsequent data pre-processing, feature extraction and pattern recognition. The results corresponding to each subject are then transmitted to the integrated classifier and the final decision is generated through a voting mechanism at the decision level, while in the centralized cBCI (Li P. et al., 2022), as shown in Figure 1, subjects’ EEG information was collected individually for data pre-processing. The pre-processed EEG data from all subjects were fused together for CNN classification identification to make the final decision for the group. The model used in this study is a centralized cBCI system, which does not rely on the voting mechanism of a distributed system, and classification is performed by a CNN-based algorithm model.

A series of experiments (Wang and Jung, 2011; Li et al., 2020, Li P. et al., 2022; Song et al., 2022) demonstrate that centralized cBCI improves overall BCI performance by fusing data from multiple subjects. To further improve the accuracy of single-trial classification, this paper combines a combination of centralized cBCI data fusion and CNN classification algorithm to identify single-trial P300. The two centralized cBCI data fusions, namely parallel data fusion and serial data fusion, can increase the effective information on the temporal and spatial domains of single-trial P300, and the CNN classification algorithm can effectively extract features on P300, hence improving the total classification accuracy and stability of P300-cBCI in the small sample case.

## Materials and methods

### Introduction to source datasets

**FIGURE 2:**
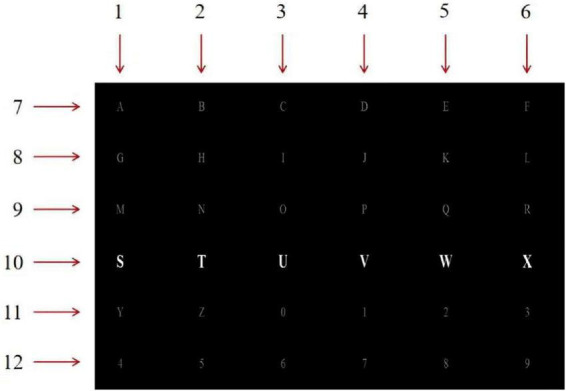
P300 speller matrix and corresponding row/column labels.

Dataset I is derived from the Kaggle dataset, which includes raw data collected by electrodes, row/column numbers flickering as stimuli, and start and end time of flickers. The experimental subjects were eight healthy participants of different ages and genders, left-handed or right-handed. The experimental data acquisition process used a standard 6 × 6 Donchin and Farewell’s P300 speller matrix stimulation interface with an interstimulus interval (ISI) of 0.125 ms. In the experiment, the acquisition channel selected eight lead channels Fz, Cz, P3, Pz, P4, PO7, PO8, Oz according to the international standard 10–20 system electrode location, and 35 alphanumeric characters were used for data acquisition. the stimulation went as follows: each row and column flickered once in a random order in one round of stimulation, so each stimulus includes 12 flickering rows/columns, and a subject was required to choose the correct row number and column number corresponding to a designated character, so as to produce 2 P300 signals. The stimulation repeated 10 times for each character, so the experimenter collected 4,200 (12*10*35) samples in total, among which 700 (2*10*35) were target stimuli. All subjects performed the same P300 stimulation evoked experiments. The stimulation interface and numbered row/column of the dataset are shown in Figure 2.

Dataset II is derived from BCI Competition III, including 50-min EEG recordings and speller matrix information of two subjects (subject A and B). One round of flickering of all the rows and columns is referred to as one trial, so each trial includes 12 row or column flickerings. Within each trial, the row or column flickers for 100 ms, with 75 ms interval between two flickering stimuli. The experiment repeats 15 times, producing 180 (12*15) row/column flickerings. The stimuli interface adopts the P300 speller matrix illustrated by Figure 2 and the corresponding row/column labels.

### Data preprocessing and fusion

The P300 EEG signal has a very low signal-to-noise ratio and mainly lies within a specific frequency range of 0.5–7.5 Hz. Collected EEG signals often include fundamental noises in various frequencies, such as industrial frequency noise, or random noise. To remove the impact of these invalid noises and improve the signal-to-noise ratio of the P300 EEG signal, an individual trial’s data extracted from a dataset are usually filtered and preprocessed with a 50 Hz trap filter and a (0.1–30 Hz) Butterworth bandpass filter. Besides the main 300 ms peak after stimulation, other peaks around it are also important, so the EEG signal in Dataset I is divided into 1 s windows using 352 timestamps to better capture key information.

Downsampling is applied on data to reduce the data transmission rate and data size. Each element value is *X*_*i,j*_, where 0 ≤ *i* ≤ N_elec_, 0 ≤ *j* ≤ N_*t*_. N_elec_ denotes the number of lead channels, N_*t*_ denotes the sampling frequency, and the sampling frequency used in the experiment is 240 Hz. The downsampling begins with data dimensionality reduction, specifically the data time domain sampling frequency is reduced from 240 Hz to 120 Hz. Then the data are normalized to prevent overfitting and avoid different data performing nearly identically in the same neural network. The calculation method is shown in formula (1).


(1) $$X_{i,j}=\frac{X_{i,j}-\bar{X}}{\sigma_{i}}$$

In Formula (1), $\bar{X}$ represents the mean value of EEG signal recorded by electrode *i* and σ_*i*_ represents the standard deviation recorded by electrode *i* (Cecotti and Graser, 2010).

**FIGURE 3:**
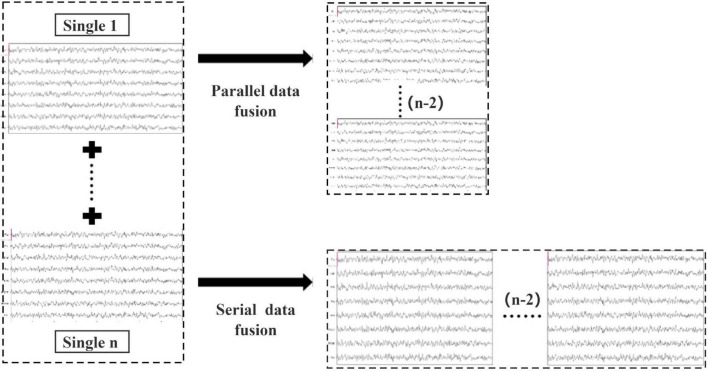
Schematic diagram of parallel data fusion and serial data fusion of multi-person data.

Two brain data fusion methods are proposed in this paper to merge the preprocessed data information in spatial and temporal domains. Specifically, both parallel data fusion and serial data fusion are performed on the data evoked by repeated identical experimental stimuli. As shown in Figure 3, n subjects labeled Single 1 to Single n were fused in two ways, and (n-2) sets of data were omitted from a total of n groups of data. Parallel data fusion increases the spatial domain feature information by fusing multi-person data stimulated by the same task, thus improving the overall performance of BCI. Serial data fusion can achieve the same goal by fusing multi-person data stimulated by the same task and adding feature information in the time domain without changing the number of leads.

### Characteristic analysis

**FIGURE 4:**
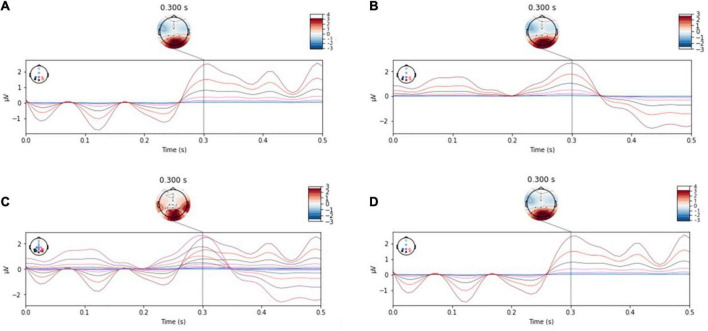
P300 characteristic distribution. **(A,B)** Single-person model. **(C)** Centralized parallel data fusion. **(D)** Centralized serial data fusion.

**FIGURE 5:**
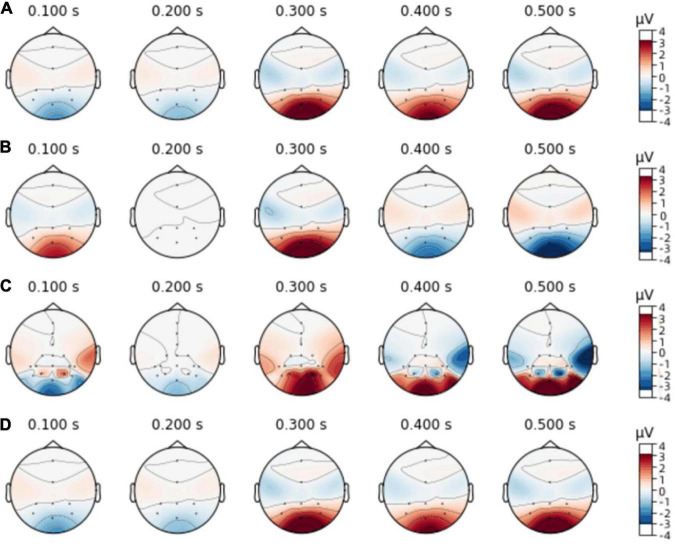
Electroencephalographic topography. **(A,B)** Single-person model. **(C)** Centralized parallel data fusion. **(D)** Centralized serial data fusion.

Two individual subjects’ data was randomly selected from data set I, which was evoked by the same stimulus experiment. Then starting with the small probability stimulus moment, the wave form during 0–500 ms after the filtered small probability stimulus evoked response was drawn, and the single-trial P300 amplitude features and EEG topographic map in single-person mode and two-person centralized data fusion mode were analyzed and compared. As shown in Figures 4, 5. In Figure 4, different colored curves in each graph correspond to different lead signals. The position of the leads is shown in the upper left corner of the diagram, the upper right corner is the amplitude color scale measured in μv, the horizontal axis represents the time and the vertical axis represents the signal amplitude of each lead. Figures 4A,B represent the EEG signals of each lead for both subjects in single-person mode. As can be seen in Figure 4, the P300 EEG signal treated with the two centralized data fusion has a more pronounced wave at around 300 ms. In this case, Figure 4C shows the centralized parallel data fusion, as the international standard 10–20 lead system was used, so by assigning the eight leads Fz, Cz, P3, Pz, P4, PO7, PO8, Oz to the eight leads FCz, CPz, CP1, CP2, P5, P6, PO3, PO4, it was possible to draw 16 lead waveforms. The increase in lead (spatial domain) information by centralized parallel data fusion is evident in Figure 4C. Figure 4D shows the centralized serial data fusion. As the centralized serial data fusion is the information added in the time domain, in terms of the lead wave crest characteristics, it is first shown as the first one of the two fusion individuals.

Figure 5 illustrates the change in amplitude corresponding to each lead position in the EEG topography in single-person mode and two-person centralized data fusion mode, with Figures 5A,B representing single-person mode, Figure 5C representing two-person centralized parallel data fusion and Figure 5D representing two-person centralized serial data fusion. It can be seen from Figures 4, 5 that this method is feasible.

### CNN classification

In this paper, Dataset I was first used, referring to the CNN structure proposed by Cecotti and Graser (2010), and the network structure parameters were adjusted based on the data characteristics of Dataset I. Taking two-person parallel data fusion as an example, the 8-Lead data set is fused into 16 leads, which increases the characteristics of lead information and spatial domain. The CNN structure is composed of Input layer, Convolution layer, Dropout layer, Maxpooling layer, Flatten layer, and Dense layer. In the CNN structure, the first and third layers are the convolutional layers, and the convolutional operation can be regarded as the inner product of the input samples and the convolutional kernel, as shown in the formula (2).


(2) $$Y_{j}^{l}=f\,\left(\sum_{i\in M_{j}}Y_{i}^{l-1}*\omega_{i\,j}^{l}+B_{j}^{l}\right)$$

In Formula (2), $Y_{j}^{l}$ is the *j* th characteristic map of the *l* th convolution layer, *f*() represents the activation function, ReLU activation function is used in this network, *M_j_* represents all input characteristic maps, $\omega_{i\,j}^{l}$ represents the convolution kernel matrix between *i* and *j*, $B_{j}^{l}$ represents offset, and * represents convolution operation.

The Dropout layer is used after the first convolution layer to prevent a decrease in sensitivity of the network model due to overfitting. The Maxpooling layer is added after the second Conv layer, which compresses the features obtained from the preceding layer with a pooling function, and selects the maximum value of all elements in each specific region of the feature map as the feature value of that region. This procedure resembles a secondary feature extraction process, retaining the main features of the data while of the data while lowering the dimensionality the dimensionality of the data, thus reducing the computational effort (He et al., 2020). So Maxpooling can effectively reduce the training parameters and over-fitting problems to form the final features. The Flatten layer is then connected with the Maxpooling layer to map the feature space calculated by the previous layer (convolution, pooling, etc.) to the sample marker space to produce the final classification result, and improve the generalization ability of the model. The specific parameters are shown in Table 1.

**TABLE 1:** Convolutional neural network (CNN) structure of two-person parallel data fusion.

Layer	Input	Type	Output	#Parameters
L1	(None,351,16,1)	Conv2D+ReLU	(None,351,16,16)	1,040
L2	(None,351,16,16)	Dropout	(None,351,16,16)	0
L3	(None,351,16,16)	Conv2D+ReLU	(None,351,16,32)	18,464
L4	(None,351,16,32)	MaxPooling2D	(None,175,8,32)	0
L5	(None,175,8,32)	Flatten	(None,44800)	0
L6	(None,44800)	Dense+ReLU	(None,64)	2,867,264
L7	(None,64)	Dense+ReLU	(None,8)	520
L8	(None,8)	Dense+softmax	(None,2)	18

Also taking two-person serial data fusion as an example, the preprocessed single-person data is fused without changing the specific data of two person. Serial data fusion is mainly carried out in the time domain. That is, the time domain information can be greatly expanded without changing the number of leads. When constructing the CNN structure of two-person serial data fusion, in order to avoid errors caused by other reasons, only the corresponding time domain parameters are changed. The CNN structure and specific parameters of two-person serial data fusion are shown in Table 2.

**TABLE 2:** Convolutional neural network (CNN) structure of two-person serial data fusion.

Layer	Input	Type	Output	#Parameters
L1	(None,702,8,1)	Conv2D+ReLU	(None,702,8,16)	1,040
L2	(None,702,8,16)	Dropout	(None,702,8,16)	0
L3	(None,702,8,16)	Conv2D+ReLU	(None,702,8,32)	18,464
L4	(None,702,8,32)	MaxPooling2D	(None,351,4,32)	0
L5	(None,351,4,32)	Flatten	(None,44928)	0
L6	(None,44928)	Dense+ReLU	(None,64)	2,875,456
L7	(None,64)	Dense+ReLU	(None,8)	520
L8	(None,8)	Dense+softmax	(None,2)	18

The ReLU function is used as the activation function of the neurons in the CNN. This method can solve the gradient vanishing problem with fast calculation speed and fast convergence speed. As shown in formula (3), when the input *x* takes a negative value, the output is 0, and when it takes a positive value, the output remains that value of *x*.


(3) R⁢e⁢L⁢U⁢(x)=m⁢a⁢x⁢(x,0)

The last layer of neurons uses the softmax function for binary classification. The function is given in formula (4) as follows, where x*_i_* is the input.


(4) $$S\,o\,f\,t\,m\,a\,x\,\left(x\right)=\frac{e^{x_{i}}}{\sum_{i}e^{x_{i}}}$$

In this paper, the CNN adopts the most robust network optimizer for the neural network. Adam, and the cross-entropy function as the loss function. The learning rate is set at 0.001, the number of trainings is set as 75, and the random mini-batch size gradient descent is set to 32, which can enable the network to be well generalized and achieve faster classification.

## Results

In order to evaluate the performance of the P300 classification algorithm, relevant evaluation criteria are considered. The standard metric for evaluating the P300 classification algorithm usually is the accuracy rate, and the formula for P300 recognition accuracy rate is given in Equation (5), which includes True Positive (*TP*), True Negative (*TN*), False Positive (*FP*), and False Negative (*FN*). TP indicates the number of samples correctly identified as positive in positive samples, *TN* indicates the number of samples correctly identified as negative in negative samples, *FP* indicates the number of samples misidentified as positive in negative samples, and *FN* indicates the number of samples misidentified as negative in positive samples (Cecotti and Graser, 2010; De Venuto and Mezzina, 2021; Liu et al., 2021).


(5) $$A\,c\,c\,u\,r\,a\,c\,y=\frac{T\,P+T\,N}{T\,P+T\,N+F\,P+F\,N}$$

The eight subjects contained in Dataset I were labeled in turn as Subjects 1–8, and their data was divided into four sets marked as C1, C2, C3, and C4, respectively, each including the data of two subjects. Then the four sets of data were used for parallel data fusion or serial data fusion. Table 3 lists the results of CNN’s single-trial P300 classification of two centralized multi-person data fusion methods and single-person mode, respectively.

**TABLE 3:** Results of convolutional neural network (CNN) single-trial P300 classification for centralized multi-person data fusion and single-person mode.

CNN	Subject	1	2	3	4	5	6	7	8	Average
	Accuracy (%)	60.72	75.00	71.43	71.43	75.00	71.43	75.00	78.57	72.32
CNN+Parallel data fusion	Subject	C1		C2		C3		C4		Average
	Accuracy (%)	78.57		82.14		85.71		85.67		83.03
CNN+Serial data fusion	Subject	C1		C2		C3		C4		Average
	Accuracy (%)	71.43		78.57		78.57		85.71		78.57

As shown in Table 3, the classification accuracy of single-trial P300 based on the fusion of two centralized multi-person data is higher than that of single-trial P300 based on the single-person mode. Specifically, the average accuracy of CNN for single-trial P300 of single-person is 71.88%, while the average classification accuracy of CNN with parallel data fusion reaches 83.03%, and that value of CNN with serial data fusion reaches 78.57%.

**FIGURE 6:**
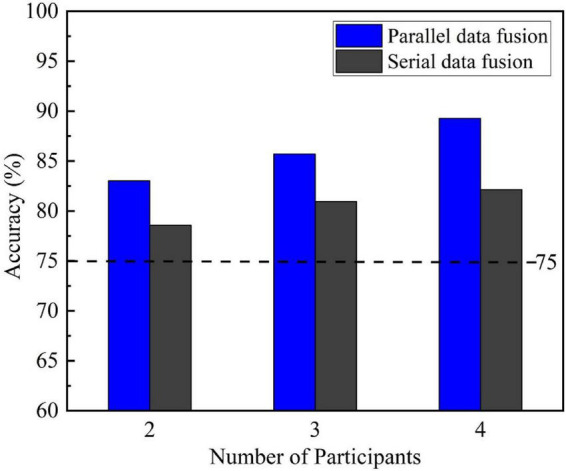
Single-trial P300 classification results of a centralized multi-person data fusion convolutional neural network (CNN) for groups with different numbers of participants.

Figure 6 compares the single-trial P300 classification results of the two data fusion methods for two-person, three-person and four-person groups and the counterpart results of the single-person mode CNN. The dotted line 75% is the highest classification accuracy of the single-trial P300 given by the single-person mode CNN. It can be seen from Figure 6, as the number of participants in the experiment increases, the average classification accuracy of the centralized multi-person data fusion method for single-trial P300 keeps improving, and both of them exceed the dotted line 75%. When the number of participants was four-person, the average classification accuracy reached 89.13% for parallel data fusion and 82.14% for serial data fusion.

In addition to accuracy, some mainstream performance metrics for binary classification problems, such as *Recall*, *Precision*, and their summed average *F1- score*, are also considered relevant for further feature recognition (Cecotti and Graser, 2010; De Venuto and Mezzina, 2021; Liu et al., 2021). The calculation formula is shown in (6), (7), and (8):


(6) $$R\,e\,c\,a\,l\,l=\frac{T\,P}{T\,P+F\,N}$$

(7) $$P\,r\,e\,c\,i\,s\,i\,o\,n=\frac{T\,P}{T\,P+F\,P}$$

(8) $$F\,1-s\,c\,o\,r\,e=2\,\frac{R\,e\,c\,a\,l\,l*P\,r\,e\,c\,i\,s\,i\,o\,n}{R\,e\,c\,a\,l\,l+P\,r\,e\,c\,i\,s\,i\,o\,n}$$

*Precision* is the proportion of genuinely positive samples in all (P300) samples that are identified as positive, and *Recall* is the proportion of positive samples that are detected from genuinely positive samples, where *Precision* and *Recall* influence each other, with both metrics being high if the detection algorithm is ideal. However, usually it is difficult to optimize both of them, when one is high the other will be low, so *F1- score* can be chosen as their combined metric. In Table 4, the bold values represent the average values of the three indicators in the single-person mode and the average values of the three indicators in the two centralized multi-person data fusion CNN for different participants. Since there are eight individual data in the data set, data fusion was carried out for four two-person groups, three three-person groups, and two four-person groups. In three-person group case, only two people were left for the last group, so one person was randomly selected from the other two groups that were already fused so as to make up three members. Then the single-trial P300 classification evaluation indicators Precision, Recall and F1- score for the two data fusion methods with two-person, three-person, and four-person groups were calculated, and the average value after centralized data fusion is taken in each case. The results are shown in Table 4. In Table 4, N-participants represent the number of participants in a group for centralized data fusion. In Table 4, N-participants represent the number of people who have undergone centralized data fusion. Since there are eight single persons in the data set, they are divided into four groups when the number of people fused is two, three groups when there are three, and two groups when there are four, and the average value after centralized data fusion is taken.

**TABLE 4:** Single-trial P300 classification results of a centralized multi-person data fusion convolutional neural network (CNN) for different numbers of participants.

Method	N-participants	Recall (%)	Precision (%)	F1- score (%)
CNN	1	**66.7**	**72.7**	**69.6**
CNN+Parallel data fusion	2	81.2	72.2	76.5
		81.2	78.5	86.7
		68.7	70.5	81.4
		75.1	75.2	85.7
	Average	**76.5**	**74.1**	**82.5**
	3	75.3	85.7	83.3
		62.5	71.4	66.7
		66.7	75.6	75.0
	Average	**68.1**	**77.5**	**75.0**
	4	51.2	66.7	70.6
		51.4	66.9	70.8
	Average	**51.3**	**66.8**	**70.7**
CNN+Serial data fusion	2	75.1	71.4	76.9
		74.5	71.2	75.6
		68.7	66.7	75.8
		62.5	66.7	76.9
	Average	**70.2**	**69.0**	**76.3**
	3	75.2	85.7	76.9
		62.5	83.3	71.4
		66.7	75.6	75.0
	Average	**68.1**	**81.5**	**74.4**
	4	51.2	66.7	70.6
		51.4	66.9	70.8
	Average	**51.3**	**66.8**	**70.7**

N-participants in Table 4 is 1, which represents the mean classification of single-trial P300 by the CNN in single-person mode. Although it can be seen from Figure 6 that the average classification accuracy of the two centralized data fusions increases as the number of participants increases, the three metrics Precision, Recall and F1-score all decrease to varying degrees as the number of participants increases. The reason behind this fact is that P300 and non-P300 data in the EEG data is unevenly distributed, even if all the recognition is made for non-P300 signals, the model can still achieve high accuracy, so the accuracy alone is not enough to achieve a scientific and persuasive evaluation, and all the four indicators should be considered comprehensively.

It can be seen from Table 4 that when the number of group member goes from 2 to 3 and 4, the recall of both centralized data fusions The highest recall rates are achieved in two-person group case with an average of 76.5% for parallel data fusion and 70.2% for serial data fusion. The F1- score averages for both centralized data fusion CNNs also reach the highest value in two-person group case, with parallel data fusion averaging 82.5% and serial data fusion averaging 76.3%. In two-person or three-person group cases, all the three metrics improved compared with those for the single-trial P300 classification in single-person mode. However, in four-person group case, the recall and precision of both centralized data fusions are slightly lower than the mean in the single-person mode, and the mean of F1- score is higher than in the single-person mode. In summary, the centralized multi-person data fusion classification algorithm has obvious advantages over the single-person mode classification algorithm. When the data of individual participants in the centralized data fusion is divided into four two-person groups, the F1–score reaches the highest when compared with the single-person mode and the number of group members is three and four, Combining the two indicators of Accuracy and F1-score, when the group members of individual participants in centralized data fusion are two, the classification single-trial P300 has the best effect. To explain the better experimental results using a data fusion group size of two compared with three and four, one possible reason could be the over-fitting of multi-dimensional data; another reason could be that the noisy nature of the EEG signal leads to saturation of the classification performance, resulting in reduced accuracy. EEG artifacts include electrode contact loosening, head movements, eye movements and muscle activity. It is known that noise levels may affect linear classification performance (Yun and Stoica, 2016).

Validation of the model on dataset I indicates that the best number of group members is two for the two centralized data fusion CNNs in single-trial P300 classification. In order to test the reproducibility of this method, this paper then applies the algorithm to the data of Dataset II. Each subject’s single-trial P300 information of 15 repeated experiments was extracted, and two subjects’ single-trial data is fused with the above-mentioned method to calculate the average classification accuracy after fusion. For consistency, the results of other advanced single-trial P300 classification algorithms analyzing the same dataset and using the same CNN structure were compared in terms of accuracy, recall, precision and F1-score. As shown in Table 5. In Table 5, bold values represent the highest two values of each column.

**TABLE 5:** Results of centralized multi-person electroencephalography (EEG) data fusion convolutional neural network (CNN) and other classification algorithms.

Method	Subject	Accuracy (%)	Recall (%)	Precision (%)	F1- score (%)
ConvLSTM (Joshi et al., 2018)	A	75.1	64.3	36.1	46.2
	B	80.2	63.4	44.0	54.0
BN3 (Liu et al., 2018)	A	73.0	63.7	33.6	44.0
	B	79.7	67.0	42.9	52.3
CNN-1 (Cecotti and Graser, 2010)	A	70.4	67.4	31.7	43.1
	B	78.2	67.8	40.7	50.9
BN3(ns)+ConvLSTM (De Venuto and Mezzina, 2021)	A	75.7	63.4	36.8	46.7
	B	82.3	65.2	47.8	**55.2**
Autoencoded CNN (De Venuto and Mezzina, 2021)	A	75.1	67.2	36.6	47.4
	B	**82.7**	66.5	**48.6**	56.2
Autoencoded CNN no LBP (De Venuto and Mezzina, 2021)	A	75.2	65.0	36.3	46.6
	B	81.7	66.3	46.6	54.7
CNN+Parallel data fusion	P_(A+B)_	**82.4**	**71.1**	**56.6**	**66.7**
CNN+Serial data fusion	S_(A+B)_	81.4	**75.1**	44.4	53.3

The comparison results in Table 5 show that the average accuracy of the CNN based on centralized parallel data fusion and serial data fusion in two-person group case reached 82.4 and 81.4%, respectively, both of which are slightly higher than that of other advanced single-trial P300 recognition algorithms. And the parallel data fusion always maintained the highest classification accuracy. In terms of recall, both algorithms based on the two centralized multi-person data fusion CNNs maintained a high level, with serial data fusion reaching around 75%. Compared with other methods, the proposed algorithms reached the higher level of accuracy, although the data was imbalanced. The two centralized brain data fusion CNNs also surpass the other algorithms in terms of F1- score, with the F1- score for parallel data fusion also maintaining the higher level. In summary, the method was shown to be reproducible. Compared with other classification algorithms, the spatial and temporal domain feature information of the single-trial P300 data layer can be increased after fusion of multi-person data, and the CNN constructed by connecting the Flatten layer with the Conv layer and Maxpooling layer can better extract and classify the features of the single-trial P300, which solves the problem of complex and time consuming operation as well as low accuracy in the process of recognizing the single-trial P300, thus achieving better recognition results.

## Discussion

The single-trial P300 classification algorithm based on centralized multi-person data fusion CNN proposed in this paper uses CNN to classify the single-trial P300 signal after centralized parallel or serial fusion of multi-person EEG data. In the CNN network structure, a Dropout layer is added after the first Conv layer to prevent overfitting, and a Maxpooling layer is used after the second Conv layer to connect the Flatten layer, extracting the maximum of all elements in each region of the convolutional layer feature map as the feature value of this region, preserving the main features of the data while reducing the dimensionality of the data. Batch Normalization is adopted to train the data in small Batch, which makes it easier to generalize the network and classify P300 signals faster. The purpose is to improve the existing multi-trial P300 classification algorithm, which is time-consuming and complex in calculation, and the single-trial P300 classification algorithm which has low accuracy. This paper uses two centralized multi-person data fusion CNN approaches to fuse the EEG data of different number of participants ranging from 2 to 4 for P300 classification. The results are evaluated with four metrics, Accuracy, Recall, Precision and F1- score, respectively, and compared with those of single-person CNN model and other advanced single-trial P300 classification algorithms, which are validated on the available public dataset Kaggle dataset and BCI Competition III. The experimental results demonstrate that the classification results of both centralized multi-person data fusion CNNs outperform the CNN classification results in single-person mode, and the four metrics of Accuracy, Recall, Precision and F1-score for detecting single-trial P300 are improved by different margins compared with other classification algorithms, so the proposed approach can achieve high accuracy in identifying single-trial P300. Comparison among the results of fusing 2, 3, and 4 people’s data as a group indicates that the best results are obtained for two-person groups.

Among the two data fusion methods used in this paper, the centralized P300-cBCI with parallel data fusion is the better choice in terms of applicability compared to the centralized P300-cBCI with serial data fusion, as it involves a smaller model and fewer training parameters. In summary, CNNs that undergo centralized two-person parallel data fusion can be more effective in improving the overall P300-cBCI classification accuracy and practical performance at small amounts of sample information. The single-trial P300 classification algorithm based on a centralized multi-person data fusion CNN proposed in this paper can be applied to online P300-cBCI systems, providing a new idea for building a more efficient P300-cBCI system, but this requires participating subjects to receive the same experimental stimuli under the same experimental conditions, and the same pre-processing of the data to be prepared for fusion. In the future, online P300-cBCI systems are to be built to enable efficient, fast and accurate classification of P300 for a number of applications, such as helping patients with text communication. This will improve the actual performance of the P300-cBCI system.

## Data availability statement

Publicly available datasets were analyzed in this study. This data can be found here: http://www.bbci.de/competition/iii/ and https://www.kaggle.com/rramele/p300samplingdataset.

## Ethics statement

Ethical review and approval were not required for the study on human participants in accordance with the local legislation and institutional requirements. Written informed consent was obtained from the individual(s) for the publication of any potentially identifiable images or data included in this article.

## Author contributions

PD: data curation, research design, data analysis, and manuscript writing. PL: conceptualization, supervision, and writing—review and editing. LC: methodology and validation. PL and PD: resources writing—original draft preparation. XL: production of figures and document retrieval. JS: data analysis and classification algorithm. All authors contributed to the article and approved the submitted version.
